# Spatiotemporal changes in microtubule dynamics during dendritic morphogenesis

**DOI:** 10.1080/19336934.2021.1976033

**Published:** 2021-10-05

**Authors:** Chun Hu, Pan Feng, Meilan Chen, Yan Tang, Peter Soba

**Affiliations:** a Key Laboratory of Brain, Cognition and Education Sciences, Ministry of Education, China, Institute for Brain Research and Rehabilitation, South China Normal University, Guangzhou, China; b Department of Ophthalmology, The Second People’s Hospital of Guangdong Province, Guangzhou, China; c Molecular Brain Physiology and Behavior, Limes Institute, University of Bonn, Bonn, Germany; d Center for Molecular Neurobiology, University Medical Center Hamburg-Eppendorf, Hamburg, Germany

**Keywords:** Microtubule, polymerization, dendritic morphogenesis, *drosophila*, neuronal development, tao kinase, spatiotemporal changes

## Abstract

Dendritic morphogenesis requires dynamic microtubules (MTs) to form a coordinated cytoskeletal network during development. Dynamic MTs are characterized by their number, polarity and speed of polymerization. Previous studies described a correlation between anterograde MT growth and terminal branch extension in *Drosophila* dendritic arborization (da) neurons, suggesting a model that anterograde MT polymerization provides a driving force for dendritic branching. We recently found that the Ste20-like kinase Tao specifically regulates dendritic branching by controlling the number of dynamic MTs in a kinase activity-dependent fashion, without affecting MT polarity or speed. This finding raises the interesting question of how MT dynamics affects dendritic morphogenesis, and if Tao kinase activity is developmentally regulated to coordinate MT dynamics and dendritic morphogenesis. We explored the possible correlation between MT dynamics and dendritic morphogenesis together with the activity changes of Tao kinase in C1da and C4da neurons during larval development. Our data show that spatiotemporal changes in the number of dynamic MTs, but not polarity or polymerization speed, correlate with dendritic branching and Tao kinase activity. Our findings suggest that Tao kinase limits dendritic branching by controlling the abundance of dynamic MTs and we propose a novel model on how regulation of MT dynamics might influence dendritic morphogenesis.

## Introduction

Developing proper dendritic morphology encompasses three major steps: ‘initiation’ of primary dendritic branches immediately after neuronal polarity is established; ‘outgrowth/guidance’ that allows primary dendrites to extend in a defined direction and distance; ‘branching’ that generates more interstitial arbours to achieve a final mature morphology, which is essential for correct information processing and functional connectivity of neuronal networks[1]. Although these steps of dendrite development are in part simultaneous, the requirement of specific molecular regulators for individual steps [2–4] implies that transition events occur during dendritic morphogenesis. How these major steps of dendritic morphogenesis are specifically regulated is however not fully eexplored.

Microtubule (MT) polymerization, i.e. the addition of tubulin dimers to the plus end of MTs, has been regarded as a critical factor in dendritic morphogenesis, because it provides mechanical and structural support, serves long-distance transport and controls local signalling events [5]. MT-based motors of the kinesin and cytoplasmic dynein families drive the transport of many types of neuronal cargoes, including organelles, synaptic vesicle precursors, neurotransmitter receptors, cell adhesion molecules, cell signalling molecules, and mRNAs [5–7]. Thus, the specific organization of the MT cytoskeleton ensures selective transport routes for the sorting of cargo into dendrites [8,9]. Importantly, MT polymerization in dendrites is not a uniform process, but finely coordinated and defined by different dynamic parameters including the number (abundance of MTs), polarity (the direction of MT polymerization in dendrites: anterograde, plus-end out; retrograde: minus-end out) and speed. Interestingly, these distinct parameters may also play separate roles, since it was reported that the number and speed of dynamic MTs affect cells division [10], spindle size [11] and directional cell migration [12], respectively. In addition, MT polarity is important for selective transport of cargoes into the axon or dendrites. It was demonstrated that the selective presence of minus-end out MTs in dendrites enables the minus-end directed motor dynein to transport cargo from the soma into dendrites [13], which is required for dendritic development [14,15]. The plus-end directed motor kinesin-1, however, has been shown to selectively transport cargo into the axon [16], despite also being present in dendrites and being required for normal dendritic development [17,18].

It was reported that more anterograde dynamic MTs are present during initial neurite outgrowth [19–21] and in short/terminal dendrites in mature neurons, while MT polymerization speed is identical in terminal and primary dendrites [22]. Loss of the MT organizer centrosomin (cnn) results in dendritic over-branching with elevated numbers of anterograde dynamic MTs [23]. In addition, previous studies showed that the presence of anterogradely polymerizing MTs in terminal dendrites correlates with terminal branch extension in *Drosophila* dendritic arborization (da) neurons [21,22], suggesting a model that anterograde MT polymerization provides a driving force for dendritic branching [23]. However, we recently found that down-regulation of *Tao* kinase in *Drosophila* da neurons resulted in an over-branched dendritic morphology with elevated numbers of dynamic MTs, but without affecting the polarity of MT polymerization [24]. Moreover, loss of the dynein light intermediate chain (*dlic2*) or Katanin-60-like (*kat60L*) function result in decreased dendritic complexity without alteration of the proportion of anterograde MTs [14,25]. In certain cases like disruption of the γ-tubulin complex, even increased anterograde MT polymerization was reported to cause dendritic under-branching [22,26]. Consequently, how MT polymerization dynamics regulates dendritic morphogenesis is still an open question. Thus, it is important to gain further insight by systematic analysis of defined dendritic development stage(s), especially in an *in vivo* system.

*Drosophila* da neurons in the larval peripheral nervous system (PNS) have served as an excellent model to investigate dendritic morphogenesis *in vivo*. Da neurons are classified from Class I (C1da) to Class IV (C4da) based on their increasing dendritic complexity [27]. Dendritic initiation and outgrowth/guidance of da neurons starts at the embryonic stage (~15–16 h after egg laying (AEL)) and branching and scaling of dendrites continue throughout larval stages in a class-specific manner [28,29]. A previous study showed an increase of minus-end MTs in dendrites from initiation to the early branching stage (~24 h AEL) in C1da neurons [19]. However, MT polymerization dynamics across larval stages have not been systematically examined so far. We thus combined imaging of dendritic development with in vivo analysis of MT dynamics throughout larval development to assess the possible contribution of different dynamic MT parameters to dendritic branching.

## Materials and methods

### Fly stocks

All fly stocks were maintained at 25°C and 70% rel. humidity on standard cornmeal/molasses food. The following alleles and transgenic lines were used: *Gal4^ppk^* (3^rd^ chromosome), *Gal4^nompC^* (3^rd^ chromosome), UAS-EB1-GFP (3^rd^ chromosome). All Drosophila stocks were obtained from Bloomington Drosophila Stock Center (Bloomington, IN).

### In vivo *confocal microscopy*

C1da and C4da neurons were imaged in live larvae by confocal microscopy at different developmental time points (Zeiss LSM700). The imaged larvae were allowed to develop to adulthood to ensure that handling and imaging did not interfere with normal development.

### Analysis of dendrite length and complexity

Dendrites of C4da and C1da neurons were traced with the Imaris Filament Tracer module (BitPlane AG) using deconvolved confocal stacks. The parameters for dendritic length and number of terminals were automatically calculated by the software. For consistency, da neurons from segments of A4-A6 were imaged and statistically analysed.

### Live imaging of EB1 dynamics

All imaging of da neurons was performed on intact larvae as previously described [25] with modifications. Neurons were imaged using a 40x oil objective on a Zeiss LSM900 confocal microscope. To avoid fluorescence bleaching and damaging of neurons, the pin hole size was increased and laser power was minimized to capture most dendrites within a single plane (without z-stack scanning). Images were recorded for about 2 min for C1da and 4m35s for C4da neurons from 24 h to 96 h AEL. In imaging experiments of C4da terminal branches, MT dynamics were recorded for 187s. Movies were analysed using ImageJ (NIH, Bethesda). EB1 comets were detected using the ImageJ Kymograph plugin and the number of EB1 tracks were quantified within the imaging period. An EB1-labelled comet was counted only if it was detectable and tracked in consecutive frames for more than 5s. It should be pointed out that unlike in C1da neurons, where we could image the complete dendritic field of individual neurons, only 1/2 (72 h AEL) or 1/3–1/4 (96 h AEL) of the C4da neuron dendritic field could be imaged at these later stages to maintain sufficient resolution and the same imaging parameters. Therefore, the presented data for number of EB1 comets in C1da is per neuron and in C4da is per 100 μm, which does not affect the overall data interpretation. The velocity of EB1 was measured using the program from http://cmci.embl.de/documents/121005advancedimg.

### pTao immunostaining

The immunostaining procedure was performed exactly as previously described [24]. Briefly, larval filets were prepared in Ringer’s buffer without calcium (130 mM NaCl, 5 mM KCl, 2 mM MgCl2,36 mM sucrose, 5 mM HEPES, pH 7.3). After 20 min fixation in 4% formaldehyde/PBS, the samples were thoroughly washed in 0.3% Triton X-100/ PBS for 3 times. The samples were incubated with methanol at −20°C for 10 min and washed briefly with 0.3% Triton X-100 in PBS for 3 times to remove methanol. The samples were transferred into blocking buffer containing 0.3% Triton X-100 in PBS with 10% normal goat serum for1h at room temperature. Anti-phospho-Tao (Ser181, 1:100, Santa Cruz Biotechnology, sc-135,712, RRID: AB_2271461) was diluted and incubated in blocking buffer overnight at 4°C. After washing, secondary Alexa dye-conjugated donkey antibodies (1:400–1000) were incubated for 1 h at room temperature. After washing with 0.3% Triton X-100 in PBS three times for 5 min, the samples were mounted in SlowFade Gold (Thermo Fisher Scientific) and prepared for imaging.

### Statistical analysis

Origin Pro (Origin Lab, Notthhampton, MA) or GraphPad Prism 7.0 were used for statistical analysis. The data were presented as mean±SD except if stated otherwise. Sample numbers are indicated in the Figure legends. One way ANOVA was used for comparing three or more groups unless stated otherwise. p < 0.05 is taken as statistically significant.

## Results

### Development of da sensory neuron dendrites across larval stages

**Figure 1.**
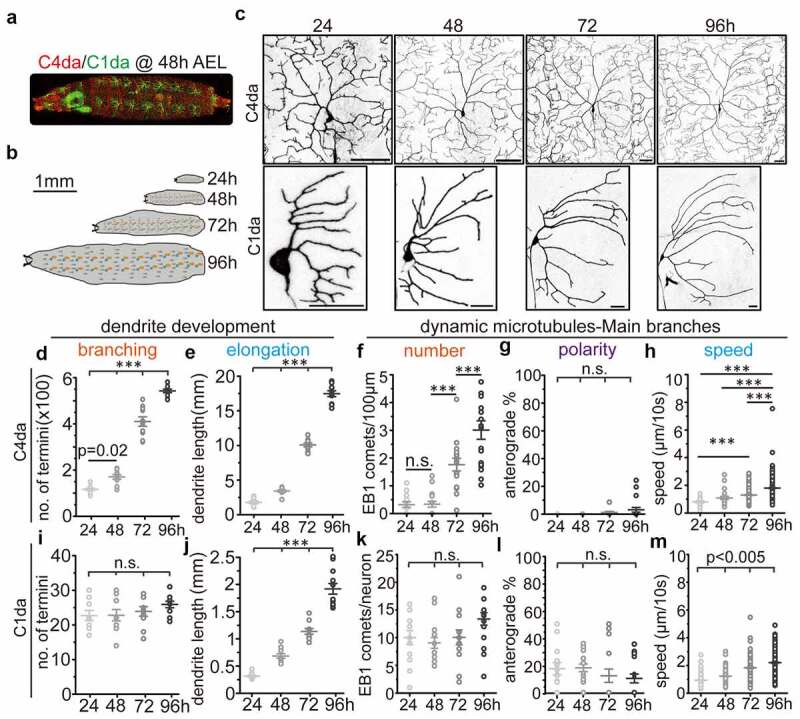
Correlation between dendritic development and MT polymerization in *Drosophila* sensory neurons at different larval stages (a) C4da neurons (red) and C1da neurons (green) segmentally distribute along the larval body wall and can be visualized by confocal microscopy during development. the overall morphology and location at 48 after egg laying (AEL) is shown. (b) schematic showing larval growth at different developmental stages. (c) representative images of C1da and C4da neurons at different larval stages (24, 48, 72 and 96 h). Scale bar: C1da: 20 µm, C4da: 50 µm. (d-e) number of dendritic terminals (d) and total dendritic length (e) of C4da neurons (ddaC). n ≥ 13/group. (f-h) the number (f), polarity (g) and speed (h) of EB1-GFP comets (dynamic MTs) in C4da neurons. n ≥ 13/group. (i-j) number of dendritic terminals (i) and total dendritic length (j) of C1da neurons (ddaD). n = 10/group. (k-m) the number (k), polarity (l) and velocity (m) of EB1-GFP comets (dynamic MTs) in C1da neurons. n = 10/group. One-way ANOVA with post-hoc bonferroni test. *p < 0.05, **p < 0.01, ***p < 0.001 as indicated. n.s.: no statistically significant difference

We first investigated how dendritic development of C1da and C4da neurons across larval stages is achieved (Figure 1(a-b)). Consistent with previous reports that C1da neurons developed virtually all arbours within a few hours during the embryonic stage [23,29,30], we did not observe obvious dendritic branching during larval stages. Instead, the existing dendrites extended continuously to scale with larval body growth (Figure 1(c,i,j)). In contrast, C4da neurons exhibit relatively simple dendritic arbours at 24 h AEL shortly after hatching, but increase the number of dendritic terminals 5-fold and their dendritic length 8-fold until 96 h AEL (Figure 1(c-e)). In addition, we noticed that the majority of new C4da neuron branches were generated from 48 h to 72 h AEL (Figure 1(d)). These data clearly show that the dendritic branching modes of C1da and C4da neurons are distinct from each other: C1da neurons exhibit virtually no and C4da neurons extremely high branching ability during larval stages. Thus, comparative analysis of C1da and C4da neuron MT dynamics should provide a useful system for detailed analysis of how cytoskeletal dynamics correlate with dendritic branching.

### Spatiotemporal changes in MT dynamics during dendritic branching

To understand the relationship between MT dynamics and dendritic branching, polymerizing MTs were tracked by *in vivo* time-lapse imaging using the plus-end binding protein EB1 tagged with GFP (EB1-GFP), which forms bright ‘comets’ marking the tip of polymerizing MTs [14,15,23,31,32]. *UAS-EB1-GFP* was expressed using *Gal4^nompC^* (C1da) or *Gal4^ppk^* (C4da) without other markers, since the background signal was strong enough to visualize the overall C1da or C4da neuron morphology as previously shown [24] (FigureS1, S2 and movies S1 and S2). For initial analysis, we focused on the main branches of C1da and C4da neurons to provide comparable conditions for assessment of MT polymerization parameters (FigureS1, S2). We then determined the number, polarity and speed of dendritic EB1 comets using kymographs (Fig. S1, S2) to ensure unbiased analysis.

In C1da neurons, the number of dynamic MTs remained unaltered from 24 h to 96 h AEL (Figure 1(k)). In addition, they displayed unchanged MT polarity during all larval stages (Figure 1(l)), but a stepwise increase in MT polymerization speed (Figure 1(m)). In C4da neurons, very few dendritic EB1 comets were visible at 24 h AEL (0.0694 ± 0.0815/100 µm×min). While no major increase in EB1 comets was detected at 48 h AEL (0.0709 ± 0.106/100 µm×min), the number of dynamic MTs was strongly increased at 72 h AEL (0.383 ± 0.200/100 µm×min) and 96 h AEL (0.654 ± 0.315/100 µm×min) (Figure 1(f)). It should be pointed out that unlike in C1da neurons, where we could image the complete dendritic field of individual neurons, only 1/2 (72 h AEL) or 1/3–1/4 (96 h AEL) of the C4da neuron dendritic field could be imaged at these later stages to maintain sufficient resolution and the same imaging parameters. Nonetheless, the absolute number of EB1 comets of individual C4da neurons (EB1 comets/neuron, estimated mean: 1.1 at 24 h, 1.8 at 48 h, 41 at 72h and 90–120 at 96 h AEL) was significantly elevated at all late time points (from 48–96 h AEL), strongly correlating with increased dendritic branching of C4da neurons during these stages (Figure 1(e)). However, we could not detect major changes in dendritic MT polarity during larval development, where less than 2% of EB1 comets displayed anterograde movement throughout all stages examined (Figure 1(g)). Consistent with our data in C1da neurons, we also found a gradual and significant increase in MT polymerization speed during C4da neuron dendrite development (Figure 1(h)). These data suggest that unlike during early dendrite initiation/outgrowth, an increase in dynamic MTs rather than the rate of anterograde polymerization correlates with dendritic branching.

**Figure 2.**
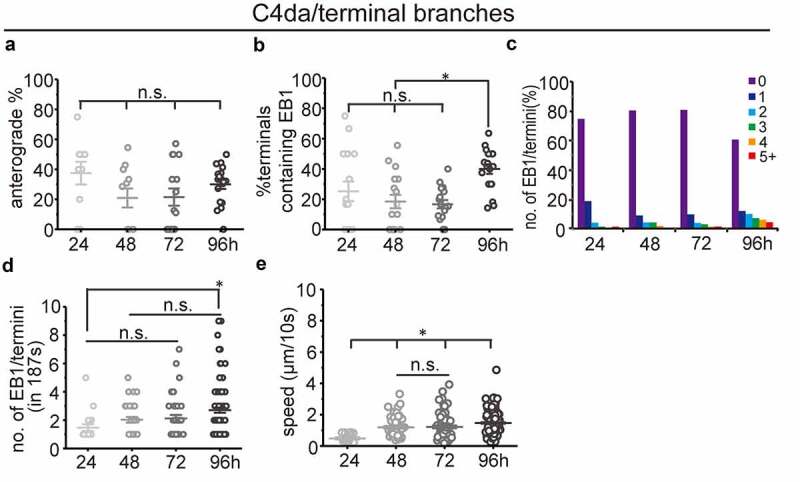
MT dynamics in terminal branches of C4da sensory neurons at different larval stages (a) percentage of anterograde dynamic MTs in terminal branches (n(branches/neurons) = 48/10, 107/11, 191/15, 250/18. neurons where no EB1 comets could be detected were excluded). (b) quantification of MT dynamics in terminal dendrites showing the percentage of terminals containing EB1 comets. (n(branches/neurons) = 76/17, 168/18, 201/17, 251/18. neurons where no EB1 comets could be detected were excluded). (c) distribution of the number of EB1 comets in terminals. note that most terminals do not contain detectable EB1 (n is the same as in (B)). (d) The average number of EB1 comets in terminal branches (n(branches/neurons) = 19/10, 33/11, 39/15, 99/18. terminals where no EB1 comets could be detected were excluded). (e) The speed of EB1 comets was measured. All data shown were analysed based on 187s of time-lapse imaging. One-way ANOVA with Bonferroni post-hoc test. n.s.: no statistical difference, *p < 0.05 as indicated

Previous studies in C4da neurons have shown increased frequencies of anterograde MT polymerization in terminal dendrites [22,33], which was suggested to be a driving force for dendritic branching. As our analysis of main branches did not confirm a correlation between dendritic branching and anterograde MT polymerization, we analysed anterograde MT polymerization in terminal branches during dendritic branching in C4da neurons. We found more anterograde MT polymerization in terminals (around 20–40%) compared to primary dendrites (<2%), which is consistent with previous reports [22,23,34]. However, we did not detect more anterograde MT polymerization in terminal branches during dendritic branching (Figure 2(a)). Instead, we found that the percentage of terminals containing EB1 comets increased only at the late dendritic branching stage (Figure 2(b), 96h AEL). We also found that the number of EB1 comets increased stepwise in terminal dendrites during dendritic branching (Figure 2(c-d)), which further confirms that the number rather than polarity of dynamic MTs is correlated with dendritic branching during larval development.

### Tao kinase activity is differentially controlled in C1da and C4da neurons during dendritic branching

**Figure 3.**
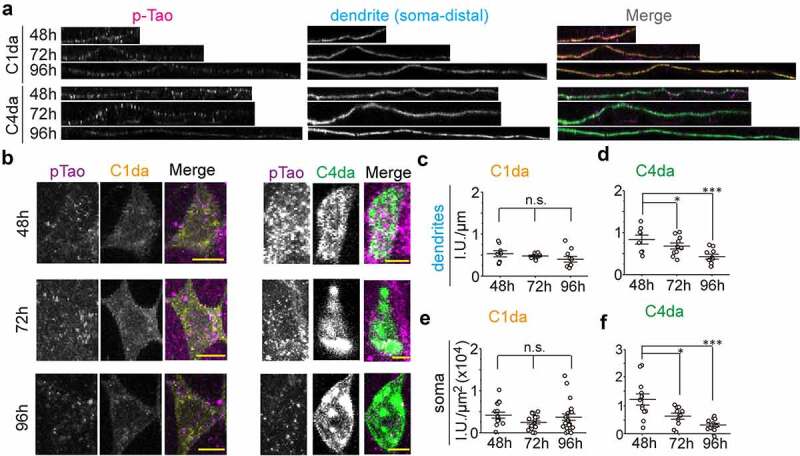
Tao kinase activity is distinctly controlled in C1da and C4da neurons. (a,b) the active form of endogenous Tao kinase was detected by anti-pTao immunostaining in dendrites (a) and somata (b) of C1da and C4da neurons during development. (c-f) quantification relative intensity of pTao signals during development in dendrites (c,d) and somata (e,f). (One-way ANOVA with bonferroni post-hoc test. *p < 0.05, ***p < 0.001 as indicated. n ≥ 8 neurons from 5 larvae for each group)

Since Tao kinase specifically regulates the number of MTs and dendritic branching in both C1da and C4da neurons in a kinase activity-dependent manner [24], we proposed that Tao kinase activity is finely tuned during development to coordinate MT dynamics and dendritic branching. To confirm this notion, we performed immunostaining of phosphorylated Tao (pTao, active form of Tao kinase) [24] in both C1da and C4da neurons at different developmental time points. We found that pTao levels are stable in C1da neurons, but gradually decrease in C4da neurons from 48 h to 96 h AEL (Figure 3). Thus, active Tao levels reflect dendritic branching activity in C1da and C4da neurons, which requires downregulation of Tao activity in C4da neurons to promote MT dynamics and branching activity. This implies a critical role for the regulation of Tao kinase activity during dendritic branching in da neurons. Thus, we speculate that Tao kinase activity is developmentally controlled by so far unknown upstream signals to maintain appropriate numbers of polymerizing MTs supporting class-specific dendritic branching patterns of da neurons.

### Perspectives on the functions of MT dynamics in dendritic morphogenesis

Previous observations from several studies based on genetic mutations with altered microtubule orientation and increased branching suggested a model that anterograde MT polymerization provides a driving force for dendritic branching [21–23]. However, studies on Tao and other genes affecting MT dynamics and dendritic morphogenesis have found no potential correlation between MT orientation and branching [14,22,24–26]. One drawback of all these studies was that they were mostly endpoint analyses that measured MT dynamics and dendritic parameters only at late stages of development. Thus, if anterograde MTs provide the driving force for dendritic branching as proposed before, we may anticipate that anterograde MTs dynamics will remain stable in C1da neurons, but dramatically increase in C4da neurons during larval development. However, our data does not support this prediction (Figure 1(g)). In addition, previous studies [14,19,33,35] and our observations showed that the higher proportion of anterograde MT polymerization in C1da neurons (around 10–20%) compared to 2–5% in C4da neurons does not correlate with dendritic branching. This further argues against a model of anterograde MT polymerization being the primary driver of dendritic branching during development.

By systematically analysing MT polymerization dynamics and dendritic branching during *Drosophila* larval development, we showed that the number instead of polarity of MT polymerization is strongly linked to dendritic branching activity. When comparing the developmental changes of dynamic MT polymerization in C1da and C4da neurons with their distinct dendritic branching profiles, dendritic branching is highly correlated with the overall number of dynamic MTs (increased in C4da vs. stable in C1da), but not their polarity (stable in both C1da and C4da) during larval development. This finding is supported by our recent findings that decreased or increased Tao kinase activity in C1da and C4da neurons results in dendritic over- or under-branching, respectively, tightly correlating with concomitantly increased or decreased numbers of dynamic MTs [24]. Strikingly, neither MT polarity nor polymerization speed were affected suggesting that indeed the number of dynamic MTs is underlying overall dendritic branching capacity. In addition, we also showed here that Tao kinase activity is differentially controlled in C1da and C4da during development, which further supports our perspective. Consistent with this notion, loss of GM130 causes dendritic under-branching and a concomitant reduction of dynamic MTs [32]. Interestingly, we observed that the speed of MT polymerization is gradually elevated in C1da and C4da neurons. Considering that C1da and C4da neurons both display dendritic extension/scaling with larval body growth, we propose that this stepwise increase in speed of MT polymerization is likely important for proportional scaling. This might be linked to increased interaction of microtubule associated proteins (MAPs), which should be further characterized by identifying molecules (e.g. AMPK [12] and Map1b [36] are likely candidates) or tools that only affect MT polymerization speed. Thus, it is a promising direction to specifically control distinct MT polymerization parameters by designing optogenetic tools, which could provide the means to test a cause-and-effect relationship for MTs in dendritic development [37].

Our data show that an overall increase in the number of dynamic MTs rather than the proposed anterograde MT polymerization correlates strongly with dendritic branching and may thus be an essential factor during development. This poses the interesting question of whether and how anterograde MT polymerization contributes to dendritic development. A recent study by Feng et al. in C1da neurons showed that the percentage of anterograde MT polymerization is much more prevalent at the dendritic initiation stage (>80%), but gradually decreases during the outgrowth stage (around 40%) [19]. Anterograde MT polymerization stays low during the early branching stage (about 20%) until dendritic maturation, although how MT polarity is altered during the transition from dendritic initiation to branching is still unknown. A reasonable hypothesis is that anterograde MT polymerization is essential for dendritic initiation, which may require much more driving force. This is partially supported by a recent study in C1da neurons showing that mutation of *centrosomin* results in more anterograde MT polymerization and dendritic branches only at the embryonic stage, when dendritic initiation and outgrowth are predominant [23]. It still should be noted that loss of *centrosomin* also elevates overall MT polymerization events [23]. Thus, the possibility that the increased number of dynamic MTs drives dendritic branching is still valid in this case.

**Figure 4.**
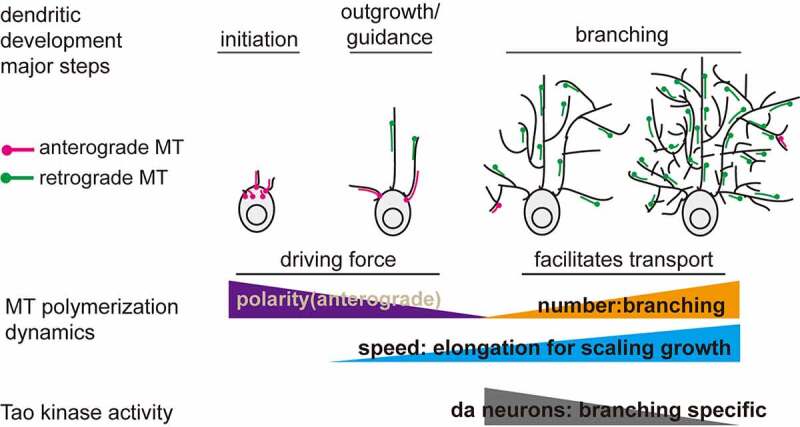
Spatiotemporal changes in MT dynamics during dendritic morphogenesis. A novel model for the function of dynamic MTs in dendritic development. Combining our current study and previous reports, we propose that anterograde MTs provide the driving force during initiation and outgrowth steps [19–21]. Higher numbers of dynamic MTs are generated due to the increased requirement of protein and membrane transport during dendritic branching [13–18] and the predominantly minus-end out MTs in main dendrites are essential to promote transport from the soma to distal dendrites via dynein [13–15]. Increased MT polymerization speed is likely linked to elongation/scaling of dendrites during larval growth due to gradually increased binding of MAPs [36]. In da neurons, Tao kinase is a core factor whose activity regulates MT dynamics during dendritic branching

Why the abundance of dynamic MTs rather than altered polarity might be linked to the dendritic branching capacity during development, at least in *Drosophila* da sensory neurons at larval stages, remains to be investigated. MTs are critical for multiple biological processes including structural maintenance, signal transduction and cargo transport. Recently, it was reported that MTs could mediate the pushing and pulling forces that contribute to membrane protrusions [5,21]. Furthermore, a reasonable possibility is that the elevated number of retrograde MTs (percentage of retrograde MTs is unaltered) may promote Dynein-dependent transport from the soma to distal dendrites, which is likely required for providing material to support dendritic branching of C4da neurons [13–15]. Conversely, the plus-end directed motor kinesin-1 has been shown to be present in dendrites [16], which may be required for retrograde cargo transport. Therefore, it is not surprising that loss of MT-based transport in dynein [14,15] and kinesin [17,18]-related proteins result in dendritic under-branching. Thus, cargo transport is likely one of the critical determinants during dendritic branching that might require the developmentally increased abundance of dynamic MTs. From a biological and developmental perspective, previous studies and our data indicate that appropriate MT polarity is likely particularly required for dendritic initiation, an increasing number of dynamic MTs for branching, and speed of MT polymerization for elongation to scale a dendritic arbour to its appropriate size and pattern (Figure 4).

MT dynamics also play an important role in dendrite pruning of *Drosophila* C4da neurons [38–44], a process where the dendritic arbour is selectively removed without causing cell death [45]. Although large-scale dendritic pruning of C4da neurons happens during metamorphosis (at early pupal stages), which is distinct from earlier dendrite development, the abundance and polarity of dynamic MTs is critical for this process. It was shown that loss of MTs starts at dendritic branch-points, the earliest local sign of degeneration during C4da neuron dendrite pruning [41,42,46]. In addition, MT polarity is also likely a major spatial determinant of degeneration, since mutations that alter dendritic MT orientation (e.g. kinesin-1/2, PP2A-Mts-Tws-Klp10A) cause a delay or block of C4da neuron dendrite pruning [38–41]. Importantly, the sites of initial neurite degeneration and the observed directionality of MT loss correlate with MT organization in neurites. In C4da neuron dendrites with their nearly uniform minus-end out MT organization, MT loss and dendrite degeneration start at proximal branch-points and expand distally until the dendrite is severed in these regions [41]. Thus, Rumpf et al. proposed a model that MT disassembly is the first local degenerative event during large-scale neurite pruning and MT polarity and organization might determine pruning initiation sites and efficiency [47].

In summary, our data and previous studies clearly highlight the importance of spatiotemporal changes of MT dynamics at distinct dendritic developmental steps including dendritic pruning, which fits and supports a model proposed for dynamic transitions of MTs in neuronal development [17]. In addition, Tao kinase is likely a core regulator of dynamic changes of MTs particularly during dendritic branching, although the mechanisms on balancing the activity of Tao kinase requires further exploration. Based on the described spatiotemporal changes of MTs in dendritic branching in *Drosophila* da neurons we propose that anterograde MTs might provide the driving force for initiation and initial outgrowth, while more MTs that are dynamic are generated during dendritic branching to support MT-based transport for the needed increase of protein and membrane delivery. Future studies will determine which cellular processes are driven by MT dynamics during different steps of dendrite development.

## Supplementary Material

Supplemental Material

## Data Availability

The data that support the findings of this study are available from the corresponding authors, CH or PS, upon reasonable request.
