# Supplementary material for: Spatiotemporal changes in microtubule dynamics during dendritic morphogenesis
Source: Fly (Austin). 2021 Oct 5;16(1):13–23. doi: 10.1080/19336934.2021.1976033 (PMC8496546; doi:10.1080/19336934.2021.1976033)
Supplement: Supplemental Material [file KFLY_A_1976033_SM0267.zip › supplementary/Supplementary data.docx]

**Supplementary data:**


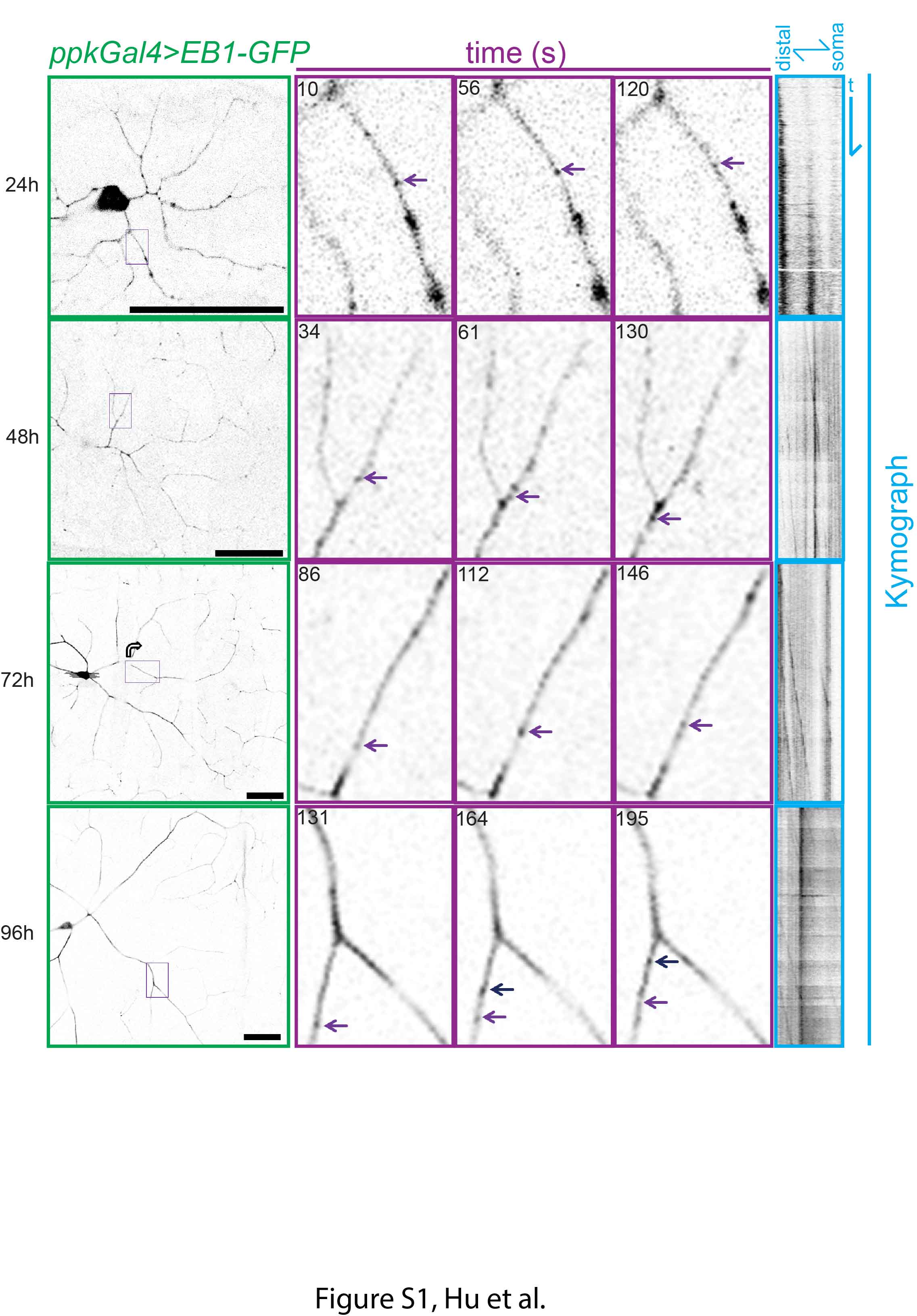


**Suppl. Fig.1 *in vivo* time-lapse analysis of EB1-GFP dynamics in C4da sensory neurons at different larval stages.**

Representative images of EB1-GFP in C4da (*ppk-Gal4>UAS-EB1-GFP*) at different larval stages. The corresponding kymograph are shown. Scale bar: 50µm.


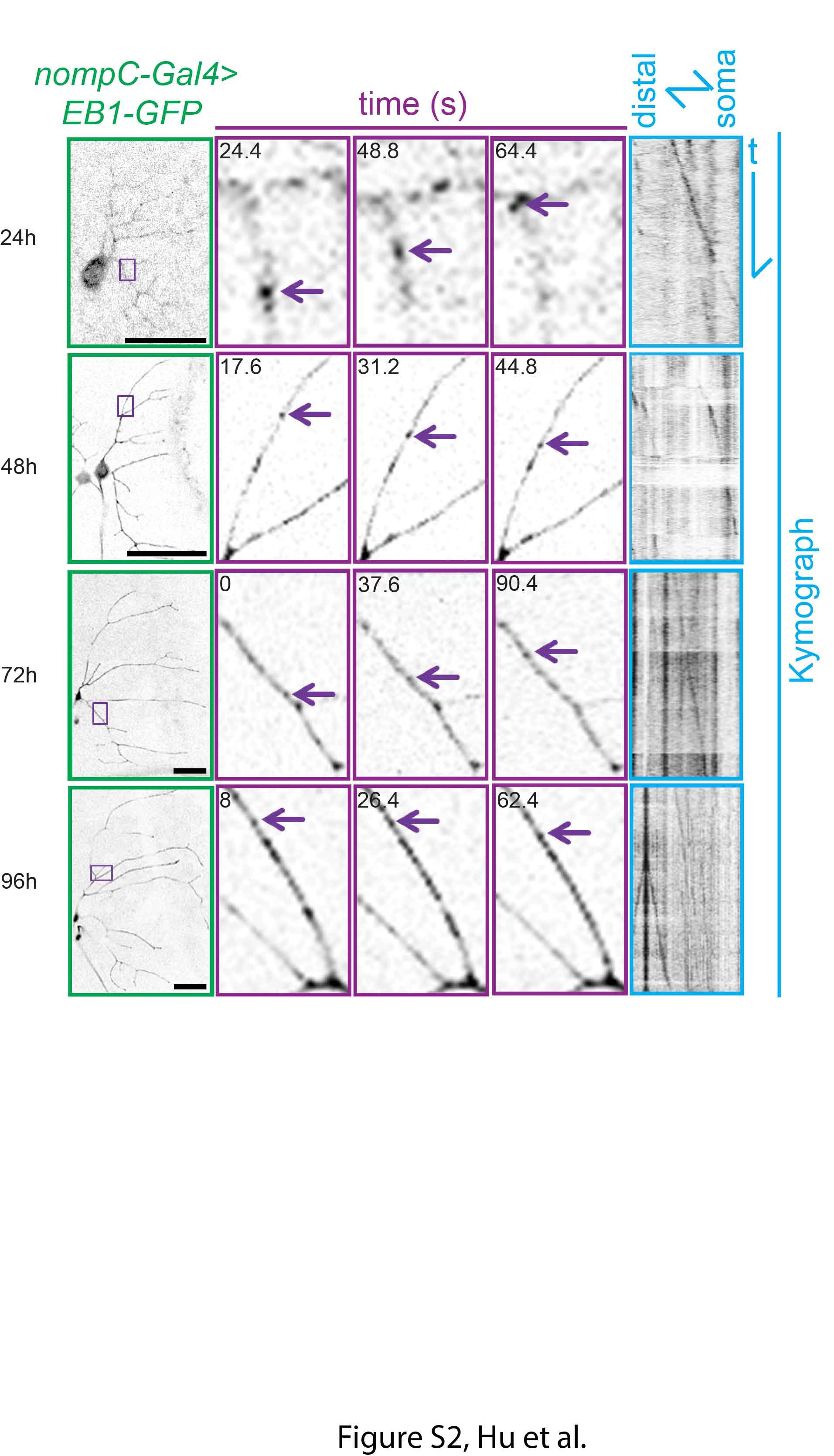


**Suppl. Fig.2 *in vivo* time-lapse analysis of EB1-GFP dynamics in C1da sensory neurons at different larval stages.**

Representative images of EB1-GFP in C1da (nompC-Gal4>UAS-EB1-GFP) at different larval stages. The corresponding kymographs are shown. Scale bar: 50µm.


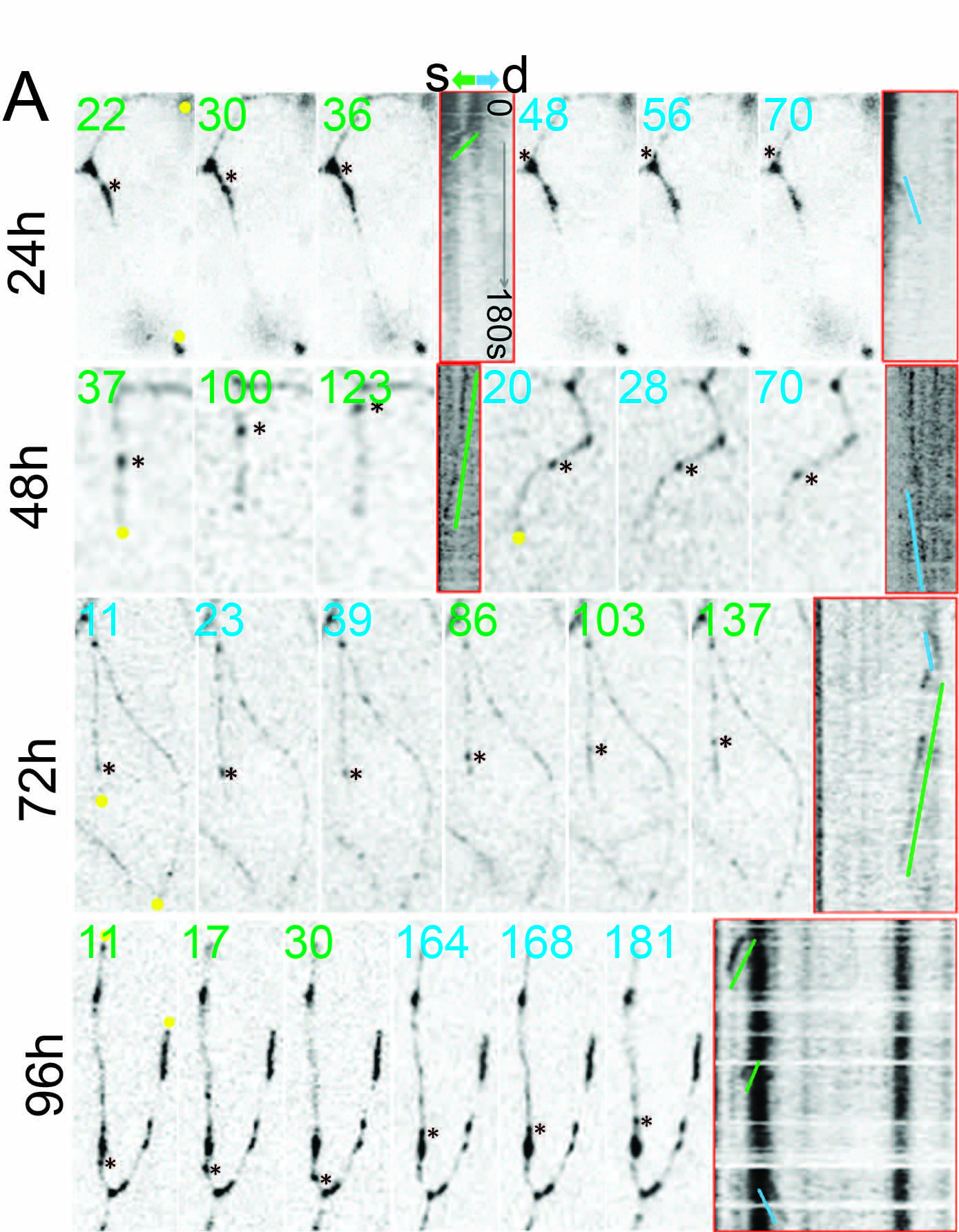


**Suppl. Fig.3 *in vivo* time-lapse analysis of EB1-GFP dynamics in terminal branches of C4da sensory neurons at different larval stages**. Yellow dots showing the end of terminal branches, stars indicate moving EB1 comets at different time points (s). The corresponding kymograph is shown next to the images. The retrograde MTs are labeled with green lines and anterograde MTs are labeled with blue lines. s: soma, d: distal. All kymographs show the total recording time of 187s. Note that at 24h and 48h of development EB1 dynamics in two different terminal branches are shown (two kymograph images), at 72h and 96h EB1 dynamics in one terminal branch are visible, which contains both anterograde and retrograde MTs (one kymograph image).

**Materials and Methods**

## Fly stocks

All fly stocks were maintained at 25°C and 70% rel. humidity on standard cornmeal/molasses food. The following alleles and transgenic lines were used: *Gal4^ppk^* (3^rd^ chromosome), *Gal4^nompC^* (3^rd^ chromosome)*, UAS-EB1-GFP* (3^rd^ chromosome). All *Drosophila* stocks were obtained from Bloomington Drosophila Stock Center (Bloomington, IN).

## *In vivo* confocal microscopy

C1da and C4da neurons were imaged in live larvae by confocal microscopy at different developmental time points (Zeiss LSM700). The imaged larvae were allowed to develop to adulthood to ensure that handling and imaging did not interfere with normal development.

## Analysis of dendrite length and complexity

Dendrites of C4da and C1da neurons were traced with the Imaris Filament Tracer module (BitPlane AG) using deconvolved confocal stacks. The parameters for dendritic length and number of terminals were automatically calculated by the software. For consistency, da neurons from segments of A4-A6 were imaged and statistically analyzed.

## Live imaging of EB1 dynamics

All imaging of da neurons was performed on intact larvae as previously described ^25^ with modifications. Neurons were imaged using a 40x oil objective on a Zeiss LSM900 confocal microscope. To avoid fluorescence bleaching and damaging of neurons, the pin hole size was increased and laser power was minimized to capture most dendrites within a single plane (without z-stack scanning). Images were recorded for about 2 min for C1da and 4m35s for C4da neurons from 24h to 96h AEL. In imaging experiments of C4da terminal branches, MT dynamics were recorded for 187s. Movies were analyzed using ImageJ (NIH, Bethesda). EB1 comets were detected using the ImageJ Kymograph plugin and the number of EB1 tracks were quantified within the imaging period. An EB1-labeled comet was counted only if it was detectable and tracked in consecutive frames for more than 5s. It should be pointed out that unlike in C1da neurons, where we could image the complete dendritic field of individual neurons, only 1/2 (72h AEL) or 1/3-1/4 (96h AEL) of the C4da neuron dendritic field could be imaged at these later stages to maintain sufficient resolution and the same imaging parameters. Therefore, the presented data for number of EB1 comets in C1da is per neuron and in C4da is per 100μm, which does not affect the overall data interpretation. The velocity of EB1 was measured using the program from <http://cmci.embl.de/documents/121005advancedimg.>

## pTao immunostaining

The immunostaining procedure was performed exactly as previously described^1^. Briefly, larval filets were prepared in Ringer’s buffer without calcium (130 mM NaCl, 5 mM KCl, 2 mM MgCl2,36 mM sucrose, 5 mM HEPES, pH 7.3). After 20 min fixation in 4% formaldehyde/PBS, the samples were thoroughly washed in 0.3% Triton X-100/ PBS for 3 times. The samples were incubated with methanol at -20°C for 10 min and washed briefly with 0.3% Triton X-100 in PBS for 3 times to remove methanol. The samples were transferred into blocking buffer containing 0.3% Triton X-100 in PBS with 10% normal goat serum for1h at room temperature. Anti-phospho-Tao (Ser181, 1:100, Santa Cruz Biotechnology, sc-135712, RRID: AB_2271461) was diluted and incubated in blocking buffer overnight at 4°C. After washing, secondary Alexa dye-conjugated donkey antibodies (1:400-1000) were incubated for 1h at room temperature. After washing with 0.3% Triton X-100 in PBS three times for 5 min, the samples were mounted in SlowFade Gold (Thermo Fisher Scientific) and prepared for imaging.

## Statistical analysis

Origin Pro (Origin Lab, Notthhampton, MA) or GraphPad Prism 7.0 were used for statistical analysis. The data were presented as mean±SD except if stated otherwise. Sample numbers are indicated in the Figure legends. One way ANOVA was used for comparing three or more groups unless stated otherwise. p<0.05 is taken as statistically significant.
