# Supplementary figures and images for: Spatiotemporal changes in microtubule dynamics during dendritic morphogenesis
Source: Fly (Austin). 2021 Oct 5;16(1):13–23. doi: 10.1080/19336934.2021.1976033 (PMC8496546; doi:10.1080/19336934.2021.1976033)

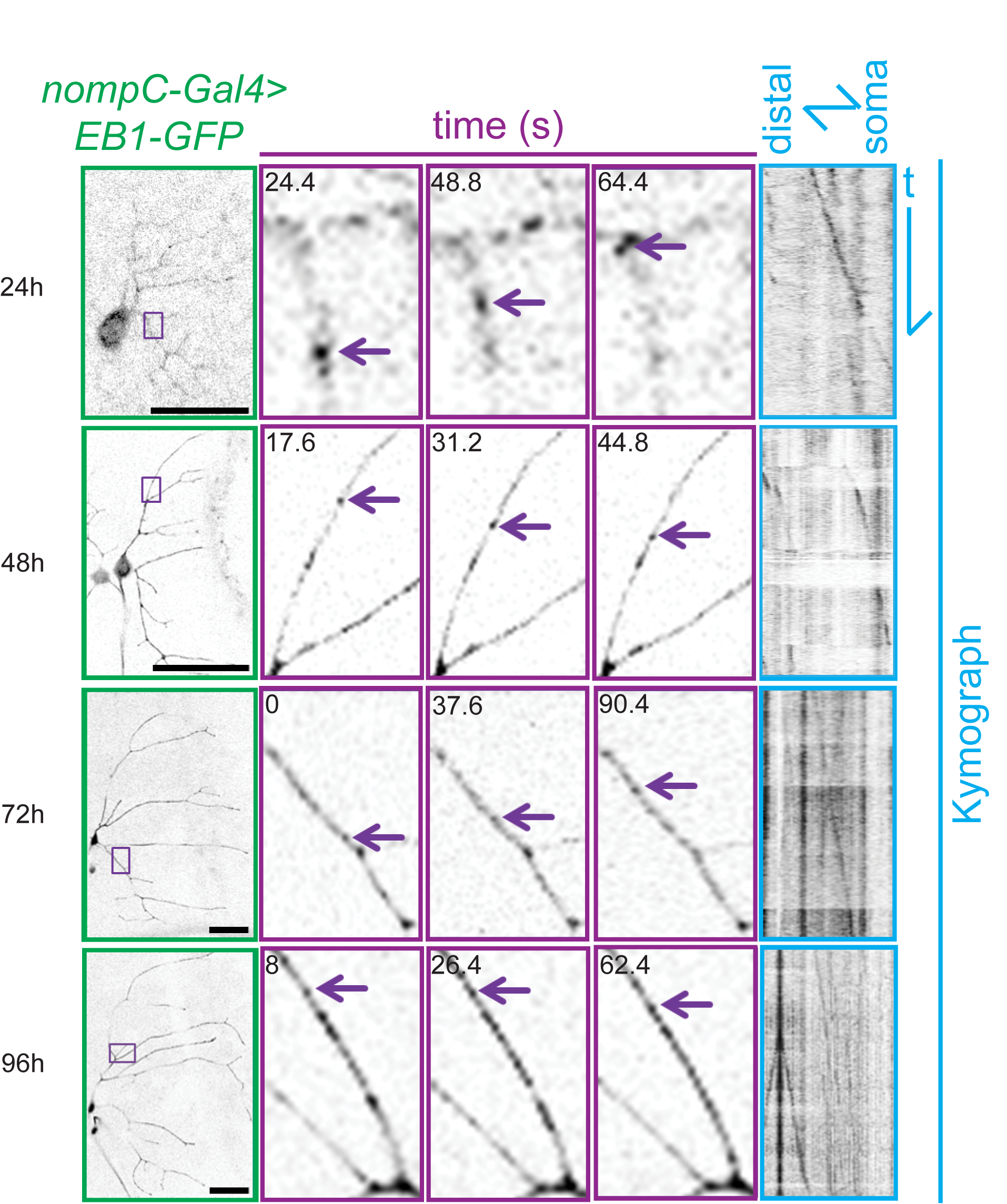

Supplement: Supplemental Material [file KFLY_A_1976033_SM0267.zip › supplementary/Suppl Fig 2 C1da EB1 dynamics.tif]

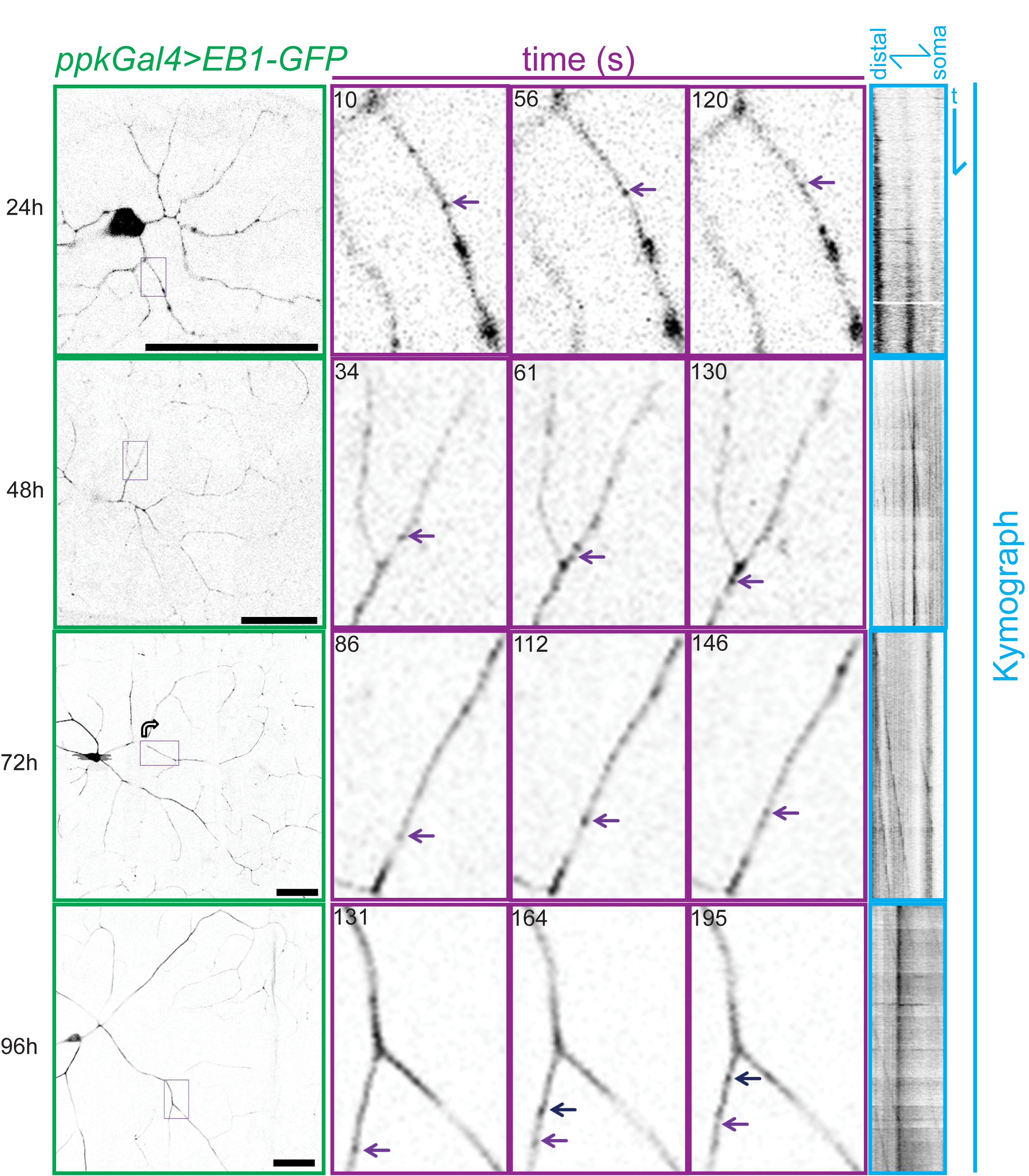

Supplement: Supplemental Material [file KFLY_A_1976033_SM0267.zip › supplementary/Suppl Fig1 C4da EB1 dynamics.tif]

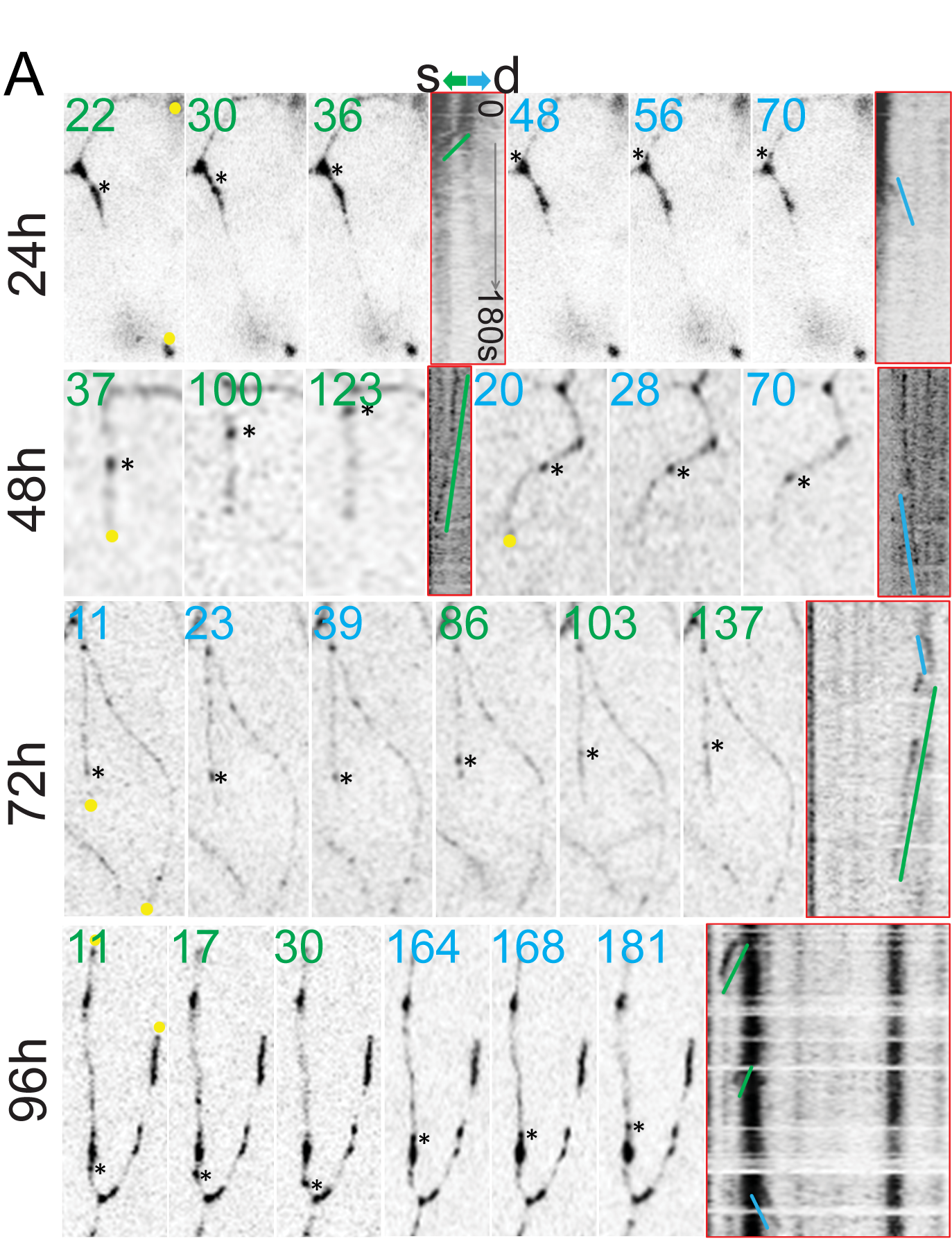

Supplement: Supplemental Material [file KFLY_A_1976033_SM0267.zip › supplementary/Suppl Fig3 C4da termianls EB1 dynamics.tif]
